# Supplementary material for: Identification of early predictors and model for bacterial infection in diabetic ketoacidosis patients: A retrospective study
Source: PLoS One. 2025 Feb 13;20(2):e0318261. doi: 10.1371/journal.pone.0318261 (PMC11825026; doi:10.1371/journal.pone.0318261)
Supplement: Attachment — Submitted filename: Reviewer feedback.docx [file pone.0318261.s002.docx]

**Reviewer feedback**

A well conceptualised study with good methodology.

Manuscript is well-written.

However, there are severe limitations especially the limited sample size.

Authors must specify the medications that affect the HPA axis in line
