# Supplementary material for: Identification of early predictors and model for bacterial infection in diabetic ketoacidosis patients: A retrospective study
Source: PLoS One. 2025 Feb 13;20(2):e0318261. doi: 10.1371/journal.pone.0318261 (PMC11825026; doi:10.1371/journal.pone.0318261)
Supplement: Attachment — Submitted filename: Response to Reviewers.docx [file pone.0318261.s003.docx]

Dear Dr. Timotius Ivan Hariyanto,

Thank you very much for your decision letter and advice on our manuscript (PONE-D-24-30892) entitled “Identification of early predictors and model for bacterial infection in diabetic ketoacidosis patients: A retrospective study”. We have revised the manuscript accordingly, and all amendments are indicated by red font in the revised manuscript. In addition, our point-by-point responses to the comments are listed below this letter.

**Replies To Reviewer 1**

1. Abstract: The type of the study is not mentioned i.e. case control study. But description of the type of the study should be mentioned.

Response: Thank you for your insightful suggestion. This study is a clinical prediction model study based on retrospective cross-sectional data, as we used historical data collected at a specific time point to develop our model. This point has been briefly mentioned in the Abstract of the revised manuscript (Page 2, Lines 22).

2. Introduction: Please revise line 43 is it 4-6% of all hospital discharge or admissions? And lines 44 and 45 for the wording, mortality rate ranging from approximately 2 to 5%.

Response: Based on the description in the original cited literature [1], the 4-9% proportion specifically refers to the percentage of all hospital discharges among patients with diabetes mellitus as the primary cause for their acute hospital admission. Correction has been made in the revised manuscript (Page 4, Line 48-50).

3. Subjects and methods: The description of the type of the study should be mentioned. Please revise line 83 and figure 1 P21 for the exclusion criteria 1) age under 18 an over 75? Line 99 please revise the methodology of Cobas e601, Roche, Switzerland. It is electrochemiluminescence (ECL) technology for immunoassay analysis.

Response: Correction has been made in the revised manuscript (Page 5, Line 86-90; Page 6, Line 107; Page 7, Line 117; Fig 1).

**Re****plies To Reviewer 2**

1. There are severe limitations especially the limited sample size.

Response: Thank you for raising this critical issue. We acknowledge the limited sample size and have addressed this limitation in our original manuscript (Page 15-16, Line 295-299). Our strict inclusion and exclusion criteria, aimed at minimizing confounding factors affecting cortisol levels in predicting DKA-associated infections, resulted in a reduced sample size. Despite this limitation, our findings provide valuable insights into early prediction of bacterial infections in DKA patients. Future studies will focus on validating this predictive model with larger sample sizes.

2. Authors must specify the medications that affect the HPA axis in line 84.

Response: Correction has been made in the revised manuscript (Page 5, Line 91; Page 6, Line 92).

**Replies To Journal Requirements**

1. Please ensure that your manuscript meets PLOS ONE's style requirements, including those for file naming.

Response: The manuscript has been carefully revised to meet all of PLOS ONE's style requirements, including those for file naming. For your convenience, all modifications have been highlighted in red font to easily identify the changes.

2. Please ensure that you have an ORCID iD and that it is validated in Editorial Manager.

Response: I confirm that my ORCID iD has been validated in Editorial Manager.

3. All PLOS journals now require all data underlying the findings described in their manuscript to be freely available to other researchers, either 1. In a public repository, 2. Within the manuscript itself, or 3. Uploaded as supplementary information.

Response: Thank you for pointing out the requirement for data availability in PLOS journals. We fully support the principles of data transparency and have taken steps to ensure compliance. We have included detailed data as supplementary information for easy access. We believe that this will facilitate reproducibility and further research based on our findings. Thank you again for your constructive input.

4. Please review your reference list to ensure that it is complete and correct. If you have cited papers that have been retracted, please include the rationale for doing so in the manuscript text, or remove these references and replace them with relevant current references.

Response:  We have thoroughly reviewed the reference list and can confirm that it is complete and correct, with no citations to any retracted papers. The author information for reference 12, which was inadvertently omitted in the original submission, has been added in the revised manuscript. This addition has been highlighted in red for easy identification.

We hope that this further revised draft of the manuscript is now acceptable for publication in your journal and look forward to hearing from you soon.

With best wishes,

Yours sincerely,

Liang Wang, Ph.D.

Corresponding author

E-mail: laiyangwangliang@163.com

**References:**

1.	Dhatariya KK, Glaser NS, Codner E, Umpierrez GE. Diabetic ketoacidosis. Nat Rev Dis Primers. 2020;6(1):40.
